# Parkinson’s Disease and Sugar Intake—Reasons for and Consequences of a Still Unclear Craving

**DOI:** 10.3390/nu14153240

**Published:** 2022-08-08

**Authors:** Julienne Haas, Daniela Berg, Anja Bosy-Westphal, Eva Schaeffer

**Affiliations:** 1 Department of Neurology, Christian-Albrechts-University, Arnold-Heller-Str. 3, 24105 Kiel, Germany; 2 Institute for Human Nutrition and Food Science, Christian-Albrechts-University, 24105 Kiel, Germany

**Keywords:** Parkinson’s disease, sugar intake, insulin metabolism, dopamine metabolism, neurodegeneration

## Abstract

Lately, studies have shown that patients with Parkinson’s disease (PD) report a strong craving for sweets and consume significantly more fast-acting carbohydrates than healthy controls. Consuming food with a high-sugar content is assumed to lead to an increase in insulin concentration, which could positively influence dopamine concentration in the brain and unconsciously be used by patients as kind of “self-medication” to compensate for a lack of dopamine in PD. On the other hand, high-sugar intake could also lead to insulin resistance and diabetes, which is discussed as a causative factor for progressive neurodegeneration in PD. In this critical appraisal, we discuss the role of sugar intake and insulin on dopamine metabolism in patients with PD and how this could influence the potential neurodegeneration mediated by insulin resistance.

## 1. Introduction

Many patients with Parkinson’s disease (PD) report a change in eating behavior with an excessive craving for sweets [1,2], sometimes already occurring before the manifestation of the cardinal motor symptoms. Lately, some studies confirmed that patients with PD prefer eating sweet foods [1,3], including chocolate [4], cakes [5] and ice cream [6]. Preferring sweet foods seems to reflect a craving or a need for fast-acting sugar more than a need for certain tastes or ingredients [7]. In fact, patients with PD consume significantly more (fast-acting) carbohydrates compared to healthy controls [1,2,8]. More precisely, they have a higher consumption of free sugar. Interestingly, a higher consumption of sugar in PD is not necessarily accompanied by an increase in weight, even in the early phases of the disease, in which increases in muscle tone, tremor or dyskinesia do not reach a degree that requires higher energy consumption [8]. In fact, many patients even lose weight [9,10]. Some studies indicate that although patients consume more fast-acting carbohydrates, there are no differences in total energy intake [1,2]. However, the reason for this change in preference has not yet been explained. Especially for patients, the effect of this change in eating habits on disease progression remains unclear. It has been suggested that a higher intake of sugar might increase dopamine (DA) concentration in the brain. Hence, increased sugar consumption in patients with PD can be seen as a form of “self-treatment” [3,11]. On the other hand, it has been shown that a high intake of fast-acting carbohydrates also affects insulin metabolism, which has lately been discussed as a factor that potentially influences progressive neurodegeneration in PD [12,13]. Therefore, it remains unclear whether increased sugar intake in PD results in benefits for the patient or, in contrast, might be a culprit triggering the progression of neurodegeneration. Taken together, the frequently observed intake of increased amounts of free sugar in patients with PD might influence pathophysiology, and thus may also hold therapeutic options.

In this critical appraisal, we give an overview of the potential mechanisms leading to increased sugar intake in patients with PD and discuss the role of insulin on DA metabolism and neurodegeneration, potentially mediated by central and peripheral insulin resistance.

## 2. Methods

To find suitable information, a search in Medline was conducted. Articles including animal and human studies, reviews and comments were identified using the terms “Parkinson’s disease” and “insulin” or “diabetes” or “diabetes mellitus” or “metformin” or “glitazones” or “glucagon-like peptide-1 receptor agonists” or “dipeptidyl peptidase 4 inhibitors” or “insulin resistance” or “sugar” or “carbohydrates” or “dopamine”. Suitable articles were also detected in the citation lists of the papers identified by the literature search. Only articles published in English up until November 2021 were evaluated in this critical appraisal.

## 3. Sugar Intake, Dopamine and Insulin in Parkinson’s Disease

### 3.1. Effects of Sugar Intake on Dopamine Concentrations in the Brain via Insulin

Primarily, the intake of sugar leads to an increase in blood glucose, which triggers insulin release in the pancreatic ß-cells (reviewed by [14]). Insulin then acts via peripheral and central insulin receptors (reviewed by [15]). In rat brains, insulin receptors are highly represented in the substantia nigra [16,17,18]. The application of intravenous glucose has been shown to lead to a transient increase in DA release in rodent substantia nigra cells [19] via several mechanisms. Thus, insulin leads to a higher firing frequency of dopaminergic neurons [20]. Additionally, insulin seems to increase the excitability of striatal cholinergic interneurons via insulin receptors, leading to an increase in striatal DA release [21]. Moreover, insulin delays the degradation of DA by reducing the expression of monoamine-oxidase (MAO). Finally, it increases DA uptake by increasing dopamine reuptake transporters (DAT) expression [22,23].

When examining post-mortem brain tissue from patients with PD, a loss of insulin receptor immunoreactivity as well as tyrosine hydroxylase protein was observed, which potentially indicates limited DA production via this pathway [24,25,26]. However, there are no further human post-mortem studies examining these possible coherences.

Following the evidence from animal models, it can be hypothesized that patients with PD unconsciously consume higher amounts of sugar to increase brain DA concentration through an insulin peak as a kind of “self-medication” to counteract the disease-related low DA concentration and consecutive symptoms [3,11]. However, it remains unclear whether an insulin peak really increases DA concentrations and thereby decreases symptoms in patients with PD as there are only few studies concerning this issue. One study showed an improvement in motor symptoms one hour after chocolate intake [7], while another study showed an association of higher sugar consumption with increased non-motor symptom burden including depression, dementia and REM behavior sleep disorder (RBD), as well as poorer quality of life in patients with PD [1]. One may argue that patients with a more severe progression of the disease, and thus more overall symptoms may crave more fast-acting sugar [1]. Conversely, higher sugar intake could lead to a more rapid disease progression. However, other studies could not confirm this association of symptom severity and increase in sugar intake [3,7]. Due to differences in study populations regarding disease duration and study design (retrospective [3] and prospective [1] observational studies, one interventional study [7]), further investigations are needed.

### 3.2. Potential Interactions between Insulin Metabolism and Neurodegeneration in PD

Although the short-term effects of high intake of sugar and increased insulin concentration in patients with PD have not been determined yet, it is essential to consider long-term effects of increased sugar consumption, as there are indices that this eating habit may be disadvantageous for patients with PD. Over time, carbohydrates with a high glycemic index are associated with inflammation, insulin resistance and diabetes [27,28,29], which are discussed as potential factors contributing to progressive neurodegeneration in PD [30]. Aside from glucose, fructose, which is often contained in the sweeteners used in processed food, has a massive impact on insulin resistance (reviewed by [31]) and needs to be studied in this respect.

In fact, several studies indicate that patients with diabetes mellitus, including type 1 and 2, have a higher risk of developing PD [32,33,34,35,36]. This risk increases with a disease duration of diabetes of more than five years [37,38]. In one study, the risk of developing PD was more pronounced in women with diabetes [39]. However, results are inconsistent as other studies could not show a higher risk of developing PD in patients with diabetes [40,41,42,43]. This can be explained in part by different study designs.

Besides these indications for an increased risk of PD in patients with diabetes, there is evidence indicating a common genetic predisposition for diabetes and PD in at least one subgroup of patients with shared genetic pathways, evidenced by several common genetic loci. One major shared pathway relates to a role in immune function, which is demonstrated by the fact that several changes occur in genes coding for the human leucocyte antigen (HLA) system. Another pathway relates to common changes in the microtubule-associated protein tau (MAPT) [44]. Additionally, patients with PD and patients with type 1 and 2 diabetes share abnormal concentrations of some microRNAs that are important for epigenetic modification (reviewed by [45]).

Finally, some studies found greater symptom severity in patients with PD and diabetes, in particular regarding postural instability, gait difficulties and a generally faster progression of motor symptoms [44,46]. While posture- and gait-related symptoms could also be caused by other diabetes-associated disorders—e.g., polyneuropathy caused by diabetes—a faster overall progression may indicate a more direct effect of insulin dysregulation on PD. The observation that patients with PD and dementia suffer more from insulin resistance compared to patients with PD without dementia is important in this respect [47]. Consistent with studies in the general population, there is evidence that patients with PD and diabetes experience greater cognitive decline and worse cognitive function [46,48]. So far, underlying mechanisms remain unclear. In this paper, the negative effects of diabetes on intracerebral small vessels and associated changes in metabolism are discussed, among other factors.

### 3.3. Insulin Pathways in the Brain

Cell studies and animal models provide evidence that insulin binds to insulin receptors in the brain, which, by phosphorylating substrates of the insulin receptor, activates two pathways associated with neuronal health: (I) the PI3K/AKT pathway, which is demonstrated to play a role in neuronal survival, reduce oxidative stress and reactive oxygen species, as well as contributing to the reduced aggregation of alpha-synuclein and (II) the MAPK pathway, fostering cell growth (reviewed by [49,50]). Supporting this, some studies showed that the inactivation of insulin receptors could lead to more proinflammatory cytokines, increased oxidative stress, as well as the aggregation of alpha-synuclein in PD rodent models [12,51], potentially contributing to progressive neuronal degeneration. Importantly, in rodents, the inactivation of insulin receptors can be caused by insulin resistance [12,52]. Consequently, the high intake of free sugar, including fructose, could contribute to insulin resistance and DA depletion in patients with PD.

On the other hand, in a PD rodent model, a depletion of dopaminergic neurons was also found to alter insulin signaling and was associated with increased markers of insulin resistance, which could lead to a vicious cycle with progressive insulin resistance and a loss of dopaminergic neurons [53].

### 3.4. Effects of Diabetes Medication on Risk of Developing PD 

As there is only little direct evidence regarding the consequences of high-sugar intake and insulin resistance in patients with PD, studies investigating the effect of antidiabetic drugs, which improve insulin resistance and thereby glucose metabolism, are worth considering. The prevalence of PD seems to vary among patients diagnosed with diabetes depending on their diabetes medication. In a retrospective cohort study with more than 100,000 patients with diabetes, individuals treated with dipeptidyl peptidase 4 (DPP4) inhibitors and glucagon-like peptide-1 (GLP-1) receptor agonists alone or in combination had a significantly lower risk of developing PD compared to patients with diabetes treated with other antidiabetics than DPP4 inhibitors, GLP-1 agonists or glitazones [54]. One other study also showed a significant reduction in PD incidence after use of DPP-4 inhibitors [55]. Moreover, treatment with insulin was also associated with a decreased risk of PD but to a smaller degree compared to DPP4 or GLP-1 agonist treatments [54]. Additionally, a meta-analysis showed a significant reduction in PD incidence in patients with diabetes treated with glitazones [56]. Conversely, three studies found no reduction in PD incidence when treated with glitazones [54,57,58]. Regarding the association of metformin and sulfonylurea with PD prevalence, treatment with sulfonylurea alone seemed to increase the risk of PD in patients with diabetes, while adding metformin seemed to lead to a decreased risk [59].

### 3.5. Effects of Diabetes Medication on Disease Progression in PD

As summarized in Table 1, many studies that investigated the effects of antidiabetics on the progression of PD used PD animal models. Rodent models are most commonly used. From these studies, causal relations explaining the associations observed in humans can be derived. Several of these studies showed the neuroprotective properties of dopaminergic neurons, less alpha-synuclein aggregation, better mitochondrial function, and anti-inflammatory as well as antioxidant effects after different antidiabetic treatments. However, only a few studies investigated antidiabetic drugs in patients with PD, with so far inconclusive results. As an example, the use of glitazones had a positive influence on disease progression in PD animal models [60,61], which could not be shown in patients with PD [62].

With regard to symptoms, several studies showed improved motor and cognitive functions in animal models using different antidiabetic drugs. In patients with PD and multiple system atrophy (MSA), an improvement of motor and cognitive function was found after application of intranasal insulin or GLP1-agonists [68,94,95,96]. Additionally, patients taking GLP-1 agonists showed increased dopamine transporter density and had a slower increase in L-Dopa use and less L-Dopa-induced dyskinesia [83].

Taken together, several antidiabetic drugs might be associated with slower symptom progression in PD, and thus may have a neuroprotective, disease-modifying, or at least symptomatic effect in PD, with randomized controlled studies still absent. However, this is only indirect evidence and does not replace the need for high-quality studies investigating the short- and long-term effects of sugar intake and insulin resistance in patients with PD.

## 4. Brain Reward Circuit—Dopamine, Insulin and Depression

Dopamine is also known for its important role in the brain reward system (reviewed by [124]), which is closely linked to depression. In patients with PD, depression is a frequent non-motor symptom and seems to be associated with the abnormal neurotransmitter release of DA and serotonin (reviewed by [125]). Remarkably, evidence showed that patients with PD and depression consume more fast-acting carbohydrates than patients with PD and without depression [1], which might indicate a higher demand of DA in the brain reward system. This is supported by the observation that healthy individuals with genetically reduced amounts of DA receptors and thereby a higher demand of DA, similar to reduced DA concentrations in patients with PD, seem to develop a “reward deficiency syndrome” and use excessive carbohydrate intake as one form of “self-medication” to balance the lack of DA [126,127,128,129].

## 5. Limitations and Future Directions

**Figure 1:**
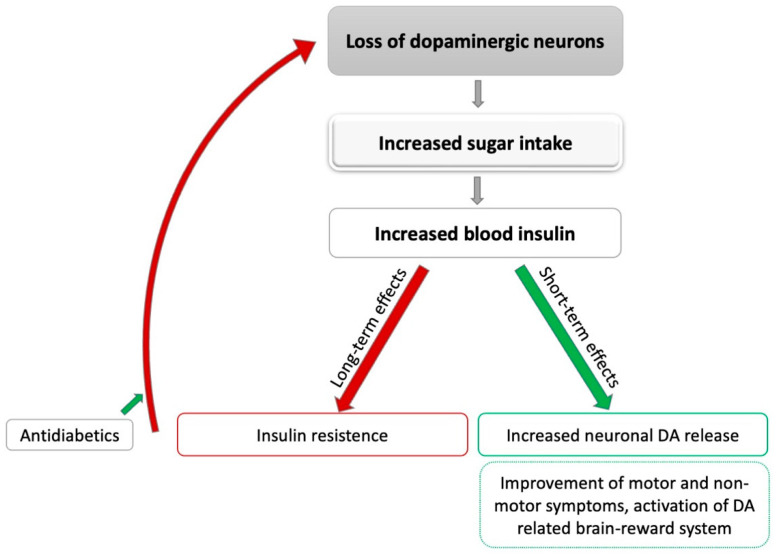
Potential effects of sugar intake on Parkinson’s disease. DA: dopamine; grey: leads to; green: positive influence; red: negative influence.

Taken together, there is some evidence from animal and cell studies that elevated insulin concentrations can lead to an increased release of DA in the brain following fast-acting carbohydrate intake. It can therefore be hypothesized that patients with PD unconsciously improve motor and potentially even non-motor symptoms by consuming high-sugar-content food (see Figure 1). However, sufficient evidence from clinical studies is still missing to confirm this assumption. On the other hand, increased insulin concentrations, following a high intake of fast-acting carbohydrates over a prolonged time, can lead to insulin resistance and diabetes, which may contribute to neuronal degeneration. Again, high-quality studies in patients with PD are still absent, especially as some of the available studies show inconclusive results. Taken together, previous studies investigating the relation between DA and insulin metabolism could not clarify whether an increased intake of high-sugar foods in PD might have the potential to improve clinical symptoms or, on the contrary, contributes to neurodegenerative processes.

Additionally, altered concentrations of insulin and insulin-growth factor (IGF) in the cerebrospinal fluid (CSF) and serum could be of interest regarding the pathophysiology of the neurodegenerative process in patients with PD. However, there are only a few studies investigating this topic. While one study could not show any differences between the insulin concentrations in the CSF of patients with PD and healthy controls [130], another study showed higher IGF-1 concentrations in the blood and CSF of patients with PD compared to the healthy individuals [131]. Moreover, one further study detected higher IGF-1 serum concentrations in patients with PD compared to healthy controls, which did not reach statistical significance [132]. Interestingly, higher IGF serum concentrations seem to be related to low concentrations of alpha-synuclein and tau in the CSF, which is assumed to represent an increased burden of those proteins in the brain tissue. As there are only a few studies investigating the topic with inconclusive results, additional research would be essential to see whether there are relevant changes in insulin and IGF-1 in the serum and CSF and whether they are related to increased concentrations of alpha-synuclein and tau in patients with PD brain tissue.

Interestingly, not every patient with PD reports an increased intake of fast-acting carbohydrates, suggesting a possible subtype of PD that is especially prone to this eating behavior. Further studies are necessary to clarify this subtype hypothesis. Longitudinally designed studies examining changes in sugar intake during disease progression, associations with symptom severity, and the possible development of insulin resistance and diabetes over time would be of high interest.

Overall, the studies discussed here have some limitations that weaken their results. Firstly, most of the studies mentioned in this critical appraisal used cell or animal models, which only offer limited transferability. Only a few studies were conducted in humans, and these studies mostly used a retrospective design. Moreover, there is a lack of more recent studies regarding insulin and DA. In fact, most studies concerning this topic are from the 1990s or early 2000s. Furthermore, it should be mentioned that there are some more aspects in patients with PD that are not fully understood yet and might contribute to changes in eating behavior. Changes in their energy expenditure and hypothalamic function, which might contribute to altered eating behavior, have been observed, among others. Especially orexin and the melanin-concentrating hormone (MCH), which are released in a homeostatic fashion, seem to be reduced in PD [133,134]. However, this seems to be somewhat correlated with a loss of appetite [135,136] and does not explain the higher intake of sugar. Additionally, changes in peripheral signals such as ghrelin and leptin concentrations have been described. However, as ghrelin and leptin regulate contradictory effects (ghrelin induces hunger, while leptin induces satiety), the relevance of these findings remains unclear [137,138]. Finally, changes in eating behavior in patients with PD could be a consequence of gastrointestinal non-motor symptoms, including dysphagia, constipation and defecatory dysfunction [139], although it is unlikely that these symptoms directly affect the intake of sugary foods.

## 6. Conclusions

In conclusion, evidence explaining the interaction of fast-acting carbohydrate intake, insulin metabolism and DA in patients with PD remains limited, and further research is needed to clarify the role of sugar intake as beneficial or harmful to patients with PD. On the one hand, there is evidence suggesting that sugar intake could improve motor and non-motor symptoms in patients with PD by increasing DA release in the short term, on the other hand, it could also lead to progressive neurodegeneration in the long term. Additionally, a high intake of fast-acting carbohydrates increases the risk for overweight and diabetes mellitus, which impairs patients’ health. Taken together, at the moment, it seems that the disadvantages of high-sugar intake predominate the benefits in the long run. Therefore, in clinical practice, it is recommended that patients are informed about the benefits of a healthy diet to positively influence the development and progression of PD and prevent other diseases [140,141]. Especially diets with a low glycemic index, rich of vitamins and polyphenols, a Mediterranean diet for example, can be recommended [142]. Moreover, patients with PD should be screened for diabetes on a regular basis, and nutrition counselling should be provided. Future research should specifically address pathophysiological mechanisms of fast-acting carbohydrates, and longitudinal observations should include the assessment of markers of carbohydrate metabolism for a better understanding of disease development, progression and, finally, the influence of therapeutic options.

## Figures and Tables

**Table 1:** Effects of antidiabetics on PD.

	Animal/Cell Model		Human	
Drug	Positive Effects	No/Negative Effects	Positive Effects	No/Negative Effects
**Intranasal insulin**	Improvement of motor function [63,64] Improvement of mitochondrial function [63,64] Improvement of cognitive function [65] Increased neuroprotection (animal model [66]; cell model [67])		Improvement of motor function [68] Improvement of cognitive function [68]	
**Metformin**	Improvement of motor function [60,69,70,71,72] Improvement of mitochondrial function [69,70,71,72,73], Increased neuroprotection [60,61,74,75,76] Decreased alpha-synuclein aggregation [61,77,78] Improvement of neuronal inflammation Increased anti-oxidant effect [71,79,80]	Increased neurodegeneration [81]		
**DPP-4** **inhibitors**	Improvement of motor function [82] Increased neuroprotection [82]		Increase in cerebral dopamine transporter [83] Slower increase in L-dopa dose [83] Less L-dopa-induced dyskinesia [83]	
**GLP-1** **agonists**	Improvement of motor function [84,85,86,87,88,89] Improvement of neuronal inflammation [84,88,90] Increased neuroprotection [84,86,88,89,90,91,92,93] Increased anti-oxidant effect [90] Decreased alpha-synuclein aggregation [88] Improvement mitochondrial function [88,93]		Improvement of cognitive function [94,95] Improvement of motor function [94,95,96]	
**GLP-1 and GIP agonists**	Improvement of motor function [97,98,99,100,101,102] Increased neuroprotection [97,98,99,100,101,102,103,104,105] Improvement of neuronal inflammation [97,98,99,104] Improvement of mitochondrial function [97]			
**Glitazones**	Reduction in glial activation [106,107,108,109,110,111] Increased neuroprotection [106,107,109,110,111,112,113,114,115,116,117,118,119] Increased anti-oxidant effect [112,120] Improvement of motor function [110,113,121,122] Improvement of neuronal inflammation [108,116,118,120,121] Improvement of cognitive function [122] Anti-depressant effect [109] Reduction in mortality [109]	Reduction in striatal dopamine through chronic treatment [115]		No effect [62]
**SGLT-2** **inhibitor**	Improvement of motor function [123] Decreased alpha-synuclein aggregation [123] Increased dopamine concentration [123] Reduction in oxidative stress [123] Improvement of neuronal inflammation [123]			

DPP-4 inhibitors, dipeptidyl peptidase 4 inhibitors, GLP-1 agonists, glicagon-like-peptide-1 agonists, GIP agonists, glucose-dependent insulinotropic polypeptide receptor agonists, SGLT-2 inhibitor, sodium-glucose transport protein 2 inhibitor.

## Data Availability

Data are available from the corresponding author upon reasonable request.
